# Molecular characterization of measles viruses that circulated in Cameroon between 2010 and 2011

**DOI:** 10.1186/1743-422X-10-71

**Published:** 2013-03-04

**Authors:** Maurice Demanou, Rajhonson Ratsitoharana, Martial Yonga, Annick Dosseh, Blanche Anya, Marie Kobela, Richard Njouom

**Affiliations:** 1 WHO National Reference Laboratory for Measles, Centre Pasteur of Cameroon, PO box 1274, Yaoundé, Cameroon; 2 WHO/IST/West Africa, Ouagadougou, Burkina Faso; 3 WHO Country Office, Yaoundé, Cameroon; 4 Expanded Program on immunization, Yaoundé, Cameroon

**Keywords:** Measles virus, Genotype B3, Nucleoprotein N, Cameroon

## Abstract

**Background:**

Measles virus (MeV) is monotypic, but genetic variation in the hemagglutinin H and nucleoprotein N genes can be analyzed by molecular epidemiologic techniques and used to study virus transmission patterns. The World Health Organization currently recognizes 8 clades (A-H) within which are 24 genotypes of MeV and one provisional genotype, d11. Genotype B3 is clearly the endemic genotype in most of African continent where it is widely distributed. We provide an update on the molecular characterization of wild-type MeVs that circulated in Cameroon between 2010 and 2011.

**Findings:**

Viral RNA was extracted directly from samples obtained from clinically diagnosed measles patients using QIAamp viral RNA Mini Kit. Reverse transcription and PCR amplification of 634 nucleotides of the N gene was performed using the SuperScript™ III One-Step. Sequence analysis of 450 of the 634 nucleotides using Clustal X 2.0 program for multiple alignments and Mega version 5 for phylogenic analysis indicated that all the viruses belonged to genotype B3 with two distinct clusters. Twenty three (77%) belonged to subgroup B3.1 and the other 7 (23%) belonged to B3.3 a recently described subtype. Circulation of cluster 3 was detected in the Far-North Region (5/7) particularly along the Chad-Cameroon border in 2010 and later in Yaounde (2/7 in Biyem-assi Health District) the capital city of Cameroon in 2011.

**Conclusion:**

This study highlights the endemic circulation in Cameroon of MeV B3 subtype 1, which probably has its source in the neighboring Nigeria, and the presence of the new subtype B3.3, suggesting a possible importation from Northern Africa where it was first described between 2008 and 2009.

## Findings

Measles virus (MeV) is a negative-sense, single-stranded RNA virus in the family *Paramyxoviridae*, genus *Morbillivirus*. Infection with this virus typically causes high fever, maculopapular rash, conjunctivitis, cough, and coryza. MeV is monotypic, but genetic variation in the hemagglutinin (H) and nucleoprotein (N) genes can be analyzed by molecular epidemiologic techniques and used to study virus transmission patterns [1]. The World Health Organization (WHO) currently recognizes 8 clades (A-H) within which are 24 genotypes of MeV and one provisional genotype, d11. Genotype B3 is clearly the endemic genotype in most of the African continent where it is widely distributed [2].

In 1998, WHO implemented measles elimination strategies and recommended that virological surveillance be established in every country. Although this surveillance is well established in all WHO regions, in some areas it still not adequate [3]. The laboratory has two main functions in measles surveillance: monitoring and verifying virus transmission (confirmation of outbreaks and identification of virus strains); and monitoring susceptibility profile of the population [4]. In Cameroon, distinct patterns of measles incidence are found in two different areas. The three northern-most Regions (Far-North, North and Adamaoua) experience major epidemics every year. Seven southern Regions show evidence of experiencing major epidemics every third year [5]. Following coincident peaks in 2000–2001 in these two cycles resulting in an increase in measles incidence countrywide, measles laboratory surveillance was established in 2001. This surveillance consisted mainly of serological confirmation of suspected cases. Following the WHO Measles and Rubella Laboratory Network initiative to expand the capacity of the national laboratories for virus isolation and detection [6], a reverse-transcription polymerase chain reaction (RT-PCR) was set up in the Centre Pasteur of Cameroon (CPC) National Reference Laboratory (NRL) for measles in early 2011. Although genetic characterization of wild-type viruses is an essential component of laboratory-base surveillance, there are very few African countries that use molecular tools for the surveillance of measles. This paper provides an update on the molecular characterization of wild-type MeVs that circulated in Cameroon between 2010 and 2011.

**Figure 1:**
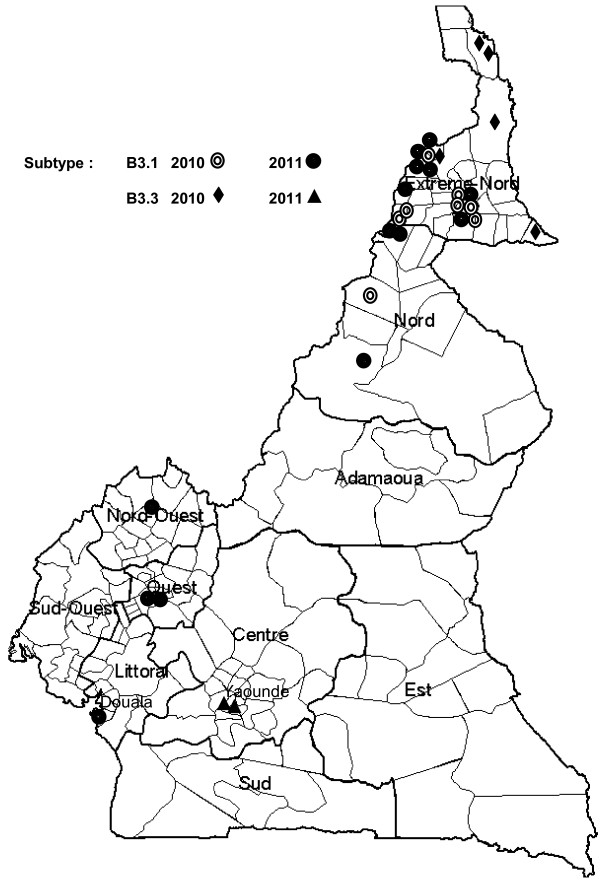
**Map of Cameroon showing the spatial (different Regions) and temporal (2010 and 2011) distribution of different subtypes of measles virus genotype B3.** An individual MeV case is represented either by a circle, a diamond or a triangle. Genotype B3 subtype 1 is represented by double circles (year 2010) and black circles (year 2011). Genotype B3 subtype 3 is represented by black diamonds (year 2010) and black triangles (year 2011).

**Figure 2:**
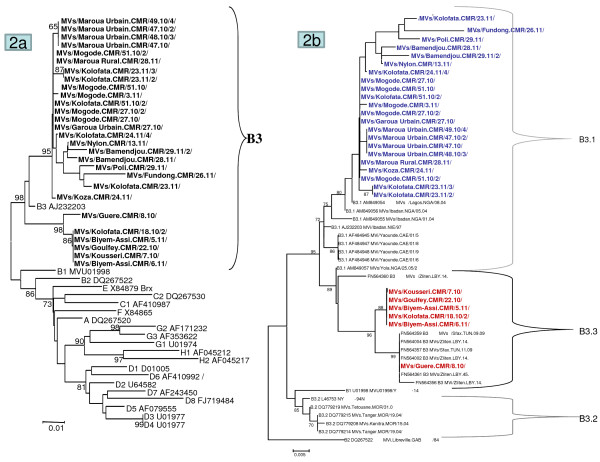
**Phylogenetic analysis of the sequences of 30 strains of MeV from Cameroon.** The trees were constructed based on the 450 nucleotides coding for the nucleoprotein N gene. These trees were prepared using Mega version 5 software and the neighbour-joining method. Bootstrap values are indicated (significant value >80%). The reference viruses are designated by their GenBank accession numbers. The Cameroonian sequences (in bold) are designated by their WHO name (in MeaNS): The comparison of the Cameroonian MeV strains with the WHO reference sequences that are recommended for genotype identification is represented on graph 2**a**. The comparison of the Cameroonian MeV genotype B3 with the sequences of other genotype B3 viruses available on GenBank is shown on graph 2**b**.

In the framework of measles case-based surveillance, an outbreak is declared in a health district when the laboratory has confirmed at least three consecutive cases of measles with positive IgM antibodies. Therefore, all clinically diagnosed cases are registered in a line listing and only throat swabs samples are collected from 5 to 10 cases by the Expanded Program on Immunization (EPI) surveillance team and sent to the NRL. From February 2010 to July 2011, measles outbreaks were registered in 37 out of 179 health districts in Cameroon and throat swabs samples were collected from patients with clinically diagnosed measles (Figure 1). Since the throat swabs were collected as part of the Ministry of Health response to the outbreaks, through the standard surveillance system as recommended by WHO and the country EPI national standard operating procedures, no authorization was required from the national ethics committee. Viral RNA was extracted directly from these samples using the commercial QIAamp viral RNA Mini Kit (QIAGEN, Germany) according to the manufacturer’s instructions. Reverse transcription and PCR amplification of the hypervariable 634 nucleotides of the carboxy-terminal of the N gene was performed using the SuperScript™ III One-Step RT-PCR System with Platinum® Taq High Fidelity (Invitrogen) and recently described reverse MeV216 (5^′^-TGGAGCTATGCCATGGGAGT-3^′^) and forward MeV214 (5^′^-TAACAATGATGGAGGGTAGG-3^′^) primers [6]. From these amplicons, a 450 nucleotide sequence (the minimum required to assign a virus to a genotype) were obtained by using BigDye terminator version 2.0 chemistry according to the manufacturer’s protocol for both sense and antisense strands on an automated ABI PRISM™ 3100 DNA Sequencer (PerkinElmer, Applied Biosystems). Sequence data were analyzed using Clustal X 2.0 program for multiple alignments and Mega version 5 for phylogenetic analysis. Dendrograms were drawn using the neighbour-joining method. The sequences reported in this article have been deposited into MeaNS (Measles Nucleotide Surveillance), the database used to track measles sequence diversity and monitor elimination of virus strains. MeV strains were named as designated by the WHO [7] (Table 1, Figure 2).

**Table 1:** Characteristics of the 30 positive throat swabs samples collected from February 2010 to July 2011 from patients with clinically diagnosed measles in Cameroon

**Laboratory code**	**Patient age (years)**	**Date of sample collection**	**Patient location (Distric-Region)**	**WHO name (in MeaNS)**
10V-2368	1.25	03/05/2010	Kolofata-Far North	MVs/Kolofata.CMR/18.10/2/
10V-5957	0.83	14/12/2010	Kolofata-Far North	MVs/Kolofata.CMR/51.10/2/
11V-11996	13.42	06/06/2011	Kolofata-Far North	MVs/Kolofata.CMR/23.11/2/
11V-11998	1.58	07/06/2011	Kolofata-Far North	MVs/Kolofata.CMR/23.11/
11V-11999	7.42	06/06/2011	Kolofata-Far North	MVs/Kolofata.CMR/23.11/3/
11V-13250	0.58	16/06/2011	Kolofata-Far North	MVs/Kolofata.CMR/24.11/4/
10V-2689	7.00	02/06/2010	Goulfey-Far North	MVs/Goulfey.CMR/22.10/
10V-2690	5.00	02/06/2010	Goulfey-Far North	-
10V-3051	9.00	04/07/2010	Mogode-Far North	MVs/Mogode.CMR/27.10/
10V-3052	2.00	04/07/2010	Mogode-Far North	MVs/Mogode.CMR/27.10/2/
11V-1040	2.00	28/12/2010	Mogode-Far North	MVs/Mogode.CMR/51.10/
11V-1041	NA	2010	Mogode-Far North	MVs/Mogode.CMR/51.10/2/
11V-1044	1.08	13/01/2011	Mogode-Far North	MVs/Mogode.CMR/3.11/
10V-425	3.00	14/02/2010	Kousseri-Far North	MVs/Kousseri.CMR/7.10/
10V-4821	1.58	25/11/2010	Maroua Urbain-Far North	MVs/Maroua Urbain.CMR/47.10/
10V-4822	1.58	25/11/2010	Maroua Urbain-Far North	MVs/Maroua Urbain.CMR/48.10/3/
10V-4824	3.33	25/11/2010	Maroua Urbain-Far North	MVs/Maroua Urbain.CMR/47.10/2/
10V-4999	6.17	02/12/2010	Maroua Urbain-Far North	MVs/Maroua Urbain.CMR/49.10/4/
10V-538	25.00	21/02/2010	Guere-Far North	MVs/Guere.CMR/8.10/
11V-13252	3.08	14/06/2011	Koza-Far North	MVs/Koza.CMR/24.11/
11V-14891	3.00	06/07/2011	Maroua Rural-Far North	MVs/Maroua Rural.CMR/28.11/
11V-14894	2.08	04/07/2011	Maroua Urbain-Far North	-
10V-3050	3.17	02/07/2010	Garoua Urbain-North	MVs/Garoua Urbain.CMR/27.10/
11V-16309	16.75	19/07/2011	Poli-North	MVs/Poli.CMR/29.11/
11V-13040	0.50	23/06/2011	Fundong-North West	MVs/Fundong.CMR/26.11/
11V-14909	0.83	13/07/2011	Bamendjou-West	MVs/Bamendjou.CMR/28.11/
11V-14939	1.50	2011	Bamendjou-West	MVs/Bamendjou.CMR/29.11/2/
11V-1038	1.00	03/02/2011	Biyem Assi-Centre	MVs/Biyem-Assi.CMR/5.11/
11V-1039	0.75	06/02/2011	Biyem Assi-Centre	MVs/Biyem-Assi.CMR/6.11/
11V-5137	3.52	30/03/2011	Nylon-Littoral	MVs/Nylon.CMR/13.11/

Throat swabs collected from seventy two patients with clinically diagnosed measles were processed. Sequences of the 3^′^ variable region of the nucleoprotein (N) gene were amplified from thirty of them. The characteristics (date of collection, age, district and region of origin) of the 30 patients are presented on Table 1. The 450 nucleotide sequences of these 30 patients were first compared to the WHO reference sequences that are recommended for genotype identification. All the sequences clustered with NY.USA 94 and Ibadan.NIE 97 [GenBank:AJ232203.1], the reference sequences for genotype B3 (Figure 2a) which is clearly the endemic genotype in most of the African continent with the exception of the North African countries in the Eastern Mediterranean Region [3]. Since genotype B3 has been divided into 3 clusters [8], the sequences from the 30 Cameroonian MeVs were compared to the sequences of other genotype B3 viruses that were available in the GenBank (Figure 2b). The results show that the Cameroonian viruses belong to two distinct clusters. Twenty three (77%) belonged to subgroup B3.1 and the other 7 (23%) belonged to B3.3. Although cluster 1 has previously been isolated in Central (Cameroon and Central African Republic), West (Ghana and Nigeria) and East (Kenya and Tanzania) African countries [9-11], cluster 2, which was not found during our study appears to be more limited to Western Africa [12]. Nevertheless, cluster 3, which was recently and for the first time, described to have circulated during 2008 and 2009 in Northern Africa (Tunisia and Libya) [8] is also present in Cameroon. This is to our knowledge, the second report of this new subtype of genotype B3 in Africa, and its first description in Central Africa. Furthermore, the Cameroonian sequences were also compared to the other B3 sequences in MeaNS (data not shown). The results of this blast search show that 77% (corresponding to the above sub-group B3.1) of the sequences were probably imported from the neighbouring Nigeria as they display more than 98% identity with the strains that circulated in Nigeria between 2010 and 2011. For the remaining 23% (B3.3), 5 of them (one from Goulfey, Kousseri and Guere and two from Biyem-Assi Health Districts) showed a 100% identity between themselves and are 99.5% identical to a strain isolated in Madrid (Spain) in 2008 from a patient with history of recent travel to Equatorial Guinea. One strain from Guere Health District exhibits a 100% identity with the 6 strains isolated in Tunisia and Libya in 2008–2009 and described as cluster 3 [8].

Circulation of cluster 3 was detected in the Far-North Region (5/7) particularly along the Chad-Cameroon border (Goulfey, Kousseri and Guere Health Districts) in 2010 and later in Yaounde (2/7 in Biyem-assi Health District) the capital city of Cameroon in 2011 (Figure 1) suggesting a probably importation of this subtype from Libya (in the Northern Africa) through Chad. It has been established after a five-year study that there are two distinct patterns of measles incidence in two different areas in Cameroon [5]. The three northern-most Regions experience major epidemics every year. Seven southern Regions show evidence of experiencing major epidemics every third year. The same patterns have been observed during the last decade and the reasons of these differences are not very clear since Cameroon is among the 31 countries with measles-containing vaccines coverage of 50-79% for infants (Vaccination coverage from WHO/UNICEF estimates 1980–2005, August 2006). Investigations should therefore be sustained to monitor the circulation of the MeV genotype B3 cluster 3 in the upcoming years in Cameroon (and in Central Africa) in order to assess its implication in multiple outbreaks faced every year especially in 2012 which extended to almost the entire country (8/10 Regions affected).

In conclusion, this study describes molecular characteristics of clade B measles viruses circulating in Cameroon with reports of recent isolates from 2010 to 2011. It also highlights the endemic circulation of MeV B3 subtype 1, which probably has its source in the neighbouring Nigeria, and the presence of the new subtype (B3.3), suggesting a possible importation from Northern Africa where it was first described between 2008 and 2009.

## Abbreviations

MeV: Measles virus;RNA: Ribonucleic acid;RT-PCR: Reverse transcription polymerase chain reaction;MeaNS: Measles nucleotide surveillance;NRL: National reference laboratory

## Competing interests

The authors declare that they have no competing interests.

## Authors’ contributions

MD designed and carried out all experimentation and drafted the manuscript. RR participated in the sequence alignment and manuscript preparation. MY assisted with data collection and experimentation. AD, BA and MK participated in the design of the study and data collection. RN assisted in phylogenic analysis and interpretation. All authors read and approved the final manuscript.

## Authors’ information

Maurice Demanou, Rajhonson Ratsitoharana, Martial Yonga and Richard Njouom belong to the international network of Pasteur institutes.
